# Alternation of Gut Microbiota in Patients with Pulmonary Tuberculosis

**DOI:** 10.3389/fphys.2017.00822

**Published:** 2017-11-17

**Authors:** Mei Luo, Yong Liu, Pengfei Wu, Dong-Xia Luo, Qun Sun, Han Zheng, Richard Hu, Stephen J. Pandol, Qing-Feng Li, Yuan-Ping Han, Yilan Zeng

**Affiliations:** ^1^Center for Growth, Metabolism and Aging, College of Life Sciences, Sichuan University, Chengdu, China; ^2^Public Health and Clinical Center of Chengdu, Chengdu, China; ^3^Olive View-UCLA Medical Center, Los Angeles, CA, United States; ^4^Cedars-Sinai Medical Center, Los Angeles, CA, United States

**Keywords:** gut microbiota, pulmonary tuberculosis, microbial diversity, *Mycobacterium tuberculosis*, new tuberculosis, recurrent tuberculosis

## Abstract

One-third of the world's population has been infected with *Mycobacterium tuberculosis* (*M. tuberculosis*), a primary pathogen of the mammalian respiratory system, while about 10% of latent infections progress to active tuberculosis (TB), indicating that host and environmental factors may determine the outcomes such as infection clearance/persistence and treatment prognosis. The gut microbiota is essential for development of host immunity, defense, nutrition and metabolic homeostasis. Thus, the pattern of gut microbiota may contribute to *M. tuberculosis* infection and prognosis. In current study we characterized the differences in gut bacterial communities in new tuberculosis patients (NTB), recurrent tuberculosis patients (RTB), and healthy control. The abundance-based coverage estimator (ACE) showed the diversity index of the gut microbiota in the patients with recurrent tuberculosis was increased significantly compared with healthy controls (*p* < 0.05). At the phyla level, Actinobacteria and Proteobacteria, which contain many pathogenic species, were significantly enriched in the feces RTB patients. Conversely, phylum Bacteroidetes, containing a variety of beneficial commensal organisms, was reduced in the patients with the recurrent tuberculosis compared to healthy controls. The Gram-negative genus *Prevotella* of oral origin from phylum of Bacteroidetes and genus *Lachnospira* from phylum of Firmicutes were significantly decreased in both the new and recurrent TB patient groups, compared with the healthy control group (*p* < 0.05). We also found that there was a positive correlation between the gut microbiota and peripheral CD4+ T cell counts in the patients. This study, for the first time, showed associations between gut microbiota with tuberculosis and its clinical outcomes. Maintaining eubiosis, namely homeostasis of gut microbiota, may be beneficial for host recovery and prevention of recurrence of *M. tuberculosis* infection.

## Introduction

Tuberculosis (TB) is a chronic potentially fatal infectious disease caused by *Mycobacterium tuberculosis* (*M. tuberculosis*). Approximately one-third of the world population has been infected with *M. tuberculosis*. While most of the initially infected individuals recover from infection through immune clearance, about only 5–10% develop active/chronic tuberculosis, implying that host and environmental factors are critical in determining the outcomes of *M. tuberculosis* infection, including drug resistance and recurrence (Tuberculosis Programme and World Health Organization, 2015). Other environmental factors make people more susceptible to tuberculosis infection. One important risk factor globally is human immunodeficiency virus (HIV), which generates immunosuppression and tolerance for the *M. tuberculosis*. Malnutrition, poverty, under privileged communities also contribute to the high incidence of active *M. tuberculosis* infections, again showing critical roles of environmental factors in the patho-physiological development for infection/transmission and prognosis of *M. tuberculosis*. Thus, identification of other environmental factors is valuable for treatment and prevention of tuberculosis infection, persistence, and recurrence.

The gut microbiome or intestinal flora is essential for the host through variety of biological functions. For instance, well established evidence shows that gut microbiome is critical for (1) development of host immune system, (2) harvesting energy from diet, (3) production of vitamin B series, (4) protection against foreign microbe invasion, (5) bile acid turnover, (6) regulation of satiety, (7) and metabolic homeostasis (Macpherson and Harris, 2004; Larsen et al., 2010). Conversely, host factors, mostly through innate and adaptive immunities such as epithelial secreted defensins and IgA and mucosa in turn impact and maintain gut microbiome in homeostasis, namely “eubiosis.” On the other hand, imbalanced microbiota, called “dysbiosis,” promotes a variety of degenerative diseases including obesity, diabetes, fatty liver diseases, cirrhosis, and certain cancers (Backhed et al., 2005; Larsen et al., 2010; Qin et al., 2014).

About 40% of the total body pool of lymphocytes are located in the intestine, suggesting a key role of the gut microbiota in generating the immune system and functionality (Grice and Segre, 2012). Since the gut microbiome is critical for the metabolic homeostasis of the host, we reasoned that the gut microbiota may impact on *M. tuberculosis* infection and prognosis. Here, we report initial findings showing the alternation of gut microbiota and association with tuberculosis prognosis among the new and recurrent patients. The findings from this work may lead to a better understanding of the role of gut microbiota in outcomes of tuberculosis and may provide new methods for tuberculosis treatments.

## Methods

### Ethics statement

This study protocol was approved by the Ethics Review Committee of the Chengdu Public Health and Therapeutic Center. Written informed consent was obtained from all participants.

### Patient information

Pulmonary tuberculosis was diagnosed using the tuberculosis diagnosis and treatment guidelines (2013, China), which include the imaging examination, clinical symptoms, physical signs, laboratory tests, medical history, progress notes and other complications.

### Study design and patient samples

A total of 37 pulmonary tuberculosis patients (19 NTB and 18 RTB) and 20 healthy controls were initially enrolled from July 2015 to June 2016. We divided the tuberculosis patients into two groups according to the following criteria. New tuberculosis group (NTB) is defined for the subjects with newly developed pulmonary tuberculosis, which had been no more than 1-week anti-tuberculosis treatment. Recurrent tuberculosis group (RTB) was defined as pulmonary tuberculosis patients who had previously been treated and declared as cured prior to becoming once again bacteriologically positive. The healthy control group consisted of 20 healthy individuals who had passed tuberculosis tests without evidence of tuberculosis or other diseases. Exclusion criteria included a history of antibiotic or probiotic treatment more than 1 week within the previous 8 weeks. All participants provided written informed consent prior to the study. Information was collected from participants included age, sex, height, weight, underlying diseases, clinical manifestations, and laboratory and radiological findings.

### Fecal sample collection and microbe DNA extraction

Fresh stool samples (200 mg/aliquot) were immediately frozen on collection and stored at −80°C before analysis. DNA from fecal samples was extracted using OMEGA stool DNA mini kit (OMEGA, China).

### High-throughput sequencing analysis

The 16S ribosomal RNA (rDNA) gene was analyzed by using IlluminaMiseq (Novogene Bioinformatics Technology Co., Ltd., USA). DNA was diluted to 1 ng/μL using sterile water. The 16S rRNA genes of distinct regions (16S V4) were amplified used specific primer 515F (5′-GTGCCAGCMGCCGCGGTAA- 3′) and 806R (5′-GGACTACHVGGGTWTCTAAT-3′) with the barcode. All PCR reactions were carried out with Phusion® High-Fidelity PCR Master Mix (New England Biolabs, USA). Samples of PCR products were mixed 1:1 (vol:vol) with loading buffer (contained SYBR green) followed by performing electrophoresis on 2% agarose gel. Samples with illuminated with SYBR green between 400 and 450 bp were chosen for further experiments. The PCR products were purified with Qiagen Gel Extraction Kit (Qiagen, Germany). Sequencing libraries were generated usingTruSeq® DNA PCR-Free Sample Preparation kit (Illumina, USA) following manufacturer's recommendations with index codes. The quality of library was assessed by Qubit@ 2.0 Fluorometer and Agilent Bioanalyzer 2100 system (Thermo Scientific, USA). Then, the library was sequenced on an IlluminaHiSeq2500 platform and 250 bp paired-end reads were generated.

### CD4^+^ T cell measurement

Whole blood samples were consecutively collected by venipuncture from the subjects. CD4^+^ T-cell enumeration was performed on CyFlow Counter (Beckman Coulter FC_500, USA) and data was analyzed by CXP analysis software.

### Bioinformatics analysis and statistics

Sequence analyses were performed by Uparse software (Uparse v7.0.1001, http://drive5.com/uparse/). Sequences with ≥97% similarity were assigned to the same Operational Taxonomy Units (OTUs). The representative sequence for each OTU was screened for further annotation. For each representative sequence, the GreenGene Database (http://greengenes.lbl.gov/Download/OTUs/) was used based on Ribosomal Database Project (RDP) classifier (Version 2.2, http://sourceforge.net/projects/rdp-classifier/) for algorithm to annotate taxonomic information. The abundance information was determined using a standard of sequence number corresponding to the sample with the least sequences. Alpha diversity in the samples was calculated with QIIME (Version 1.7.0). Beta diversity (Chao1 or Simpson index or Shannon index) analysis was used to evaluate differences of samples in species complexity. Beta diversities on both weighted and un-weighted unifrac were calculated by QIIME software. PCoA analysis was displayed by WGCNA package.

The Mann-Whitney test was used to evaluate the difference of BMI between the patients and the control group (GraphPad Prism software Version 6.01, USA). The differences in relative abundance were analyzed by the *T*-test (R software Version 2.15.3). The correlation between relative abundance and CD4^+^ T cells and specific microbes was computed using Spearman Ranking Correlation (GraphPad Prism software Version 6.01).

## Results

### Human subject characteristics

To assess the fecal microbiota in the patient groups, we initially enrolled 37 tuberculosis patients (19 patients had new tuberculosis as NTB, and 18 patients recurrent tuberculosis as RTB) and 20 healthy controls. Detailed characteristics of enrolled participants are given in Table 1. Age was similar between the tuberculosis patients (45.1y for NTB, 46.6 for RTB), but differed from the healthy controls (35.3y). There was a higher percentage of male individuals in the patient groups (male: female = 15:3). The median body mass index (BMI) was significantly lower in both the NTB group (19.1) and the RTB group (19.0), compared with the healthy individuals (22.8, Mann-Whitney test, *p* < 0.01). The sputum smear positive and sputum-culture positive rates were higher in the patients in the RTB group than that in the in the NTB group. There were 6 multi-drug resistance (MDR) patients in the RTB group, but none in the NTB group.

**Table 1:** Population characteristics.

	**TB patients**		**Health controls**
	**New tuberculosis (*n* = 19)**	**Recurrent tuberculosis (*n* = 18)**	**(*n* = 20)**
Age; median (range in years)	45.1 (14–75)	46.6 (15–77)	35.1 (25–66)
**Gender**			
Male	10	15	12
Female	9	3	8
Male/Female	1.1	5.0	1.5
Body mass index (BMI)	19.1 ± 2.6^*^	19.0 ± 1.4^*^	22.8 ± 1.5
Smear positive (%)	7 (36.8)	14 (77.8)	–
Sputum-culture positive (%)	11 (57.9)	16 (88.9)	–
**Drug Susceptibility Pattern (%)**			
MDR-TB	0	6 (33.3)	–

*Data are expressed as the mean ± SD. MDR-TB, multidrug-resistant tuberculosis. The U Mann–Whitney test was used to evaluate the two groups*.

* *P < 0.01, significant difference in comparison with the healthy control group*.

### Overview of gut bacterial diversities in the new and recurrent tuberculosis groups, and the control human subjects

To analyze the microbiota for their association with patients with new tuberculosis and the recurrent tuberculosis in comparison with that from the healthy controls, 57 fecal samples were subjected to 16S rDNA gene sequencing analysis. A total number of 1,951,408 high-quality 16S rDNA gene sequencing tags (average 52,740 per sample) were processed from 37 stool samples of patients with active tuberculosis. Among them, we identified an average of 632 OTUs (the operational taxonomic unit, which is equivalent to bacterial species) for the NTB, and an average of 675 OTUs were identified for the recurrent group. For the 20 samples in the healthy control group, we generated 1,083,132 sequencing tags (average 54,157 per sample), which gave 533 OTUs (Table 2). Thus, the results suggested that *M. tuberculosis* infection is related to the increased biodiversity of the gut microbiome in the two tuberculosis groups.

**Table 2:** 16S rRNA DNA sequencing data summary.

	**TB Patients**		**Healthy controls**
	**New tuberculosis (*n =* 19)**	**Recurrent tuberculosis (*n =* 18)**	**(*n =* 20)**
Total_tag	52,565 ± 6,905	52,925 ± 9,307	54,157 ± 6,094
Unique_tag	710 ± 356	863 ± 415	751 ± 354
OTUs	632 ± 223	675 ± 237	533 ± 126

*Data are expressed as the mean ± SD*.

**Figure 1:**
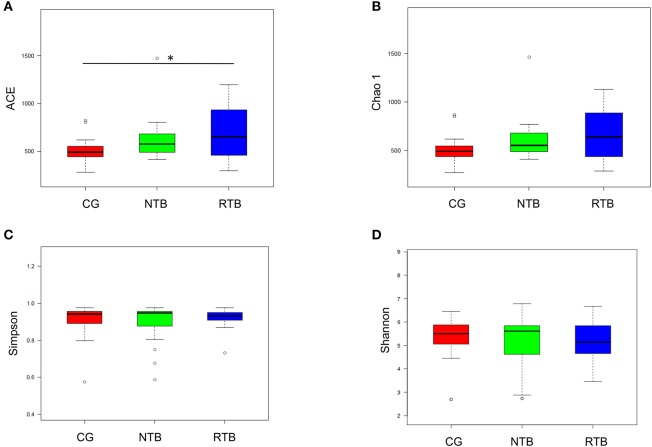
Altered biodiversity of gut microbiota in the new and recurrent tuberculosis patients in comparison with healthy control. **(A,B)** The new TB patients (NTB, *n* = 19), recurrent TB group (NTB, *n* = 18), and healthy human subjects, control (CG) are described in the Methods and Materials. The alpha-diversity, richness of gut microbes, was determined by ACE index and Chao1 index. **(C,D)** The ecological diversity of gut microbiota in the three groups of human subjects was measured by Simpson index and Shannon index. The *p*-values were calculated using Wilcox test. Statistical significance is displayed as ^*^p < 0.05.

We then thoroughly assessed the overall diversity through the parameters of the Abundance-based Coverage Estimator (ACE-diversity index), Chao1-diversity index, the Simpson index, and Shannon-diversity index. The alpha-diversity, which is the mean species diversity in gut, showed that the fecal microbiota from the recurrent tuberculosis was notably different compared to the control. The ACE-diversity index in the patients in the NTB was moderately greater than that in the control group, but the index for the recurrent group was statistically greater than that in the healthy control group (Figure 1A, Wilcox test, *p* < 0.01). The Chao1-diversity index was moderately increased in the two groups of tuberculosis patients (Figure 1B). The Simpson index was used to measure the degree of concentration when individuals were classified into types, and Shannon index was used to quantify the uncertainty (Figures 1C,D). There were no significant differences among the groups in terms of the Simpson and Shannon indexes.

**Figure 2:**
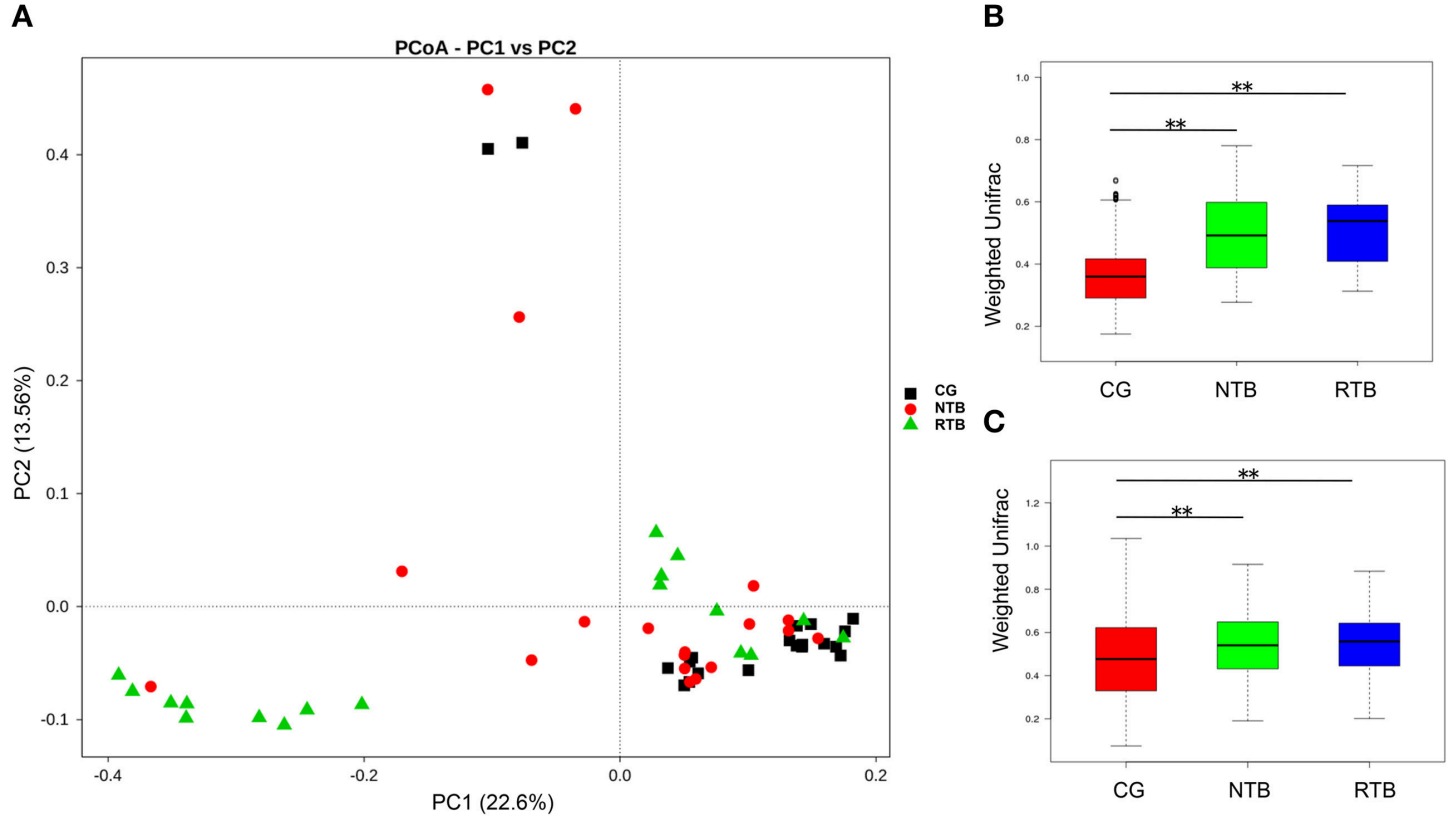
The beta-diversity of the microbial communities in the new and recurrent tuberculosis patients and the healthy control. **(A)** Principal Coordinates Analysis (PCoA) plot based on unweighted UniFrac distance. Each dot represents one sample from each group. **(B)** Beta-diversity index difference based on unweighted UniFrac. **(C)** Beta-diversity index difference based on weighted UniFrac. The *p*-values were calculated using Wilcox test. Statistical significance is displayed as ^**^p < 0.01.

The unweighted principal component analysis (unifracPCoA) indicated that the fecal microbial communities were different among the three groups. However, the pattern of microbiota in the new tuberculosis was relatively close to that of the control group, while the recurrent group had a greater difference from the new and the control groups (Figure 2A). Although a few samples overlapped, the two tuberculosis groups had significantly greater PCA variation than that in the control group (Figures 2B,C, Wilcox test, *p* < 0.01). The results from the PCA analysis are consistent with the increased alpha-diversity as shown in the Figure 1.

### The bacteria differ significantly among the three groups

**Figure 3:**
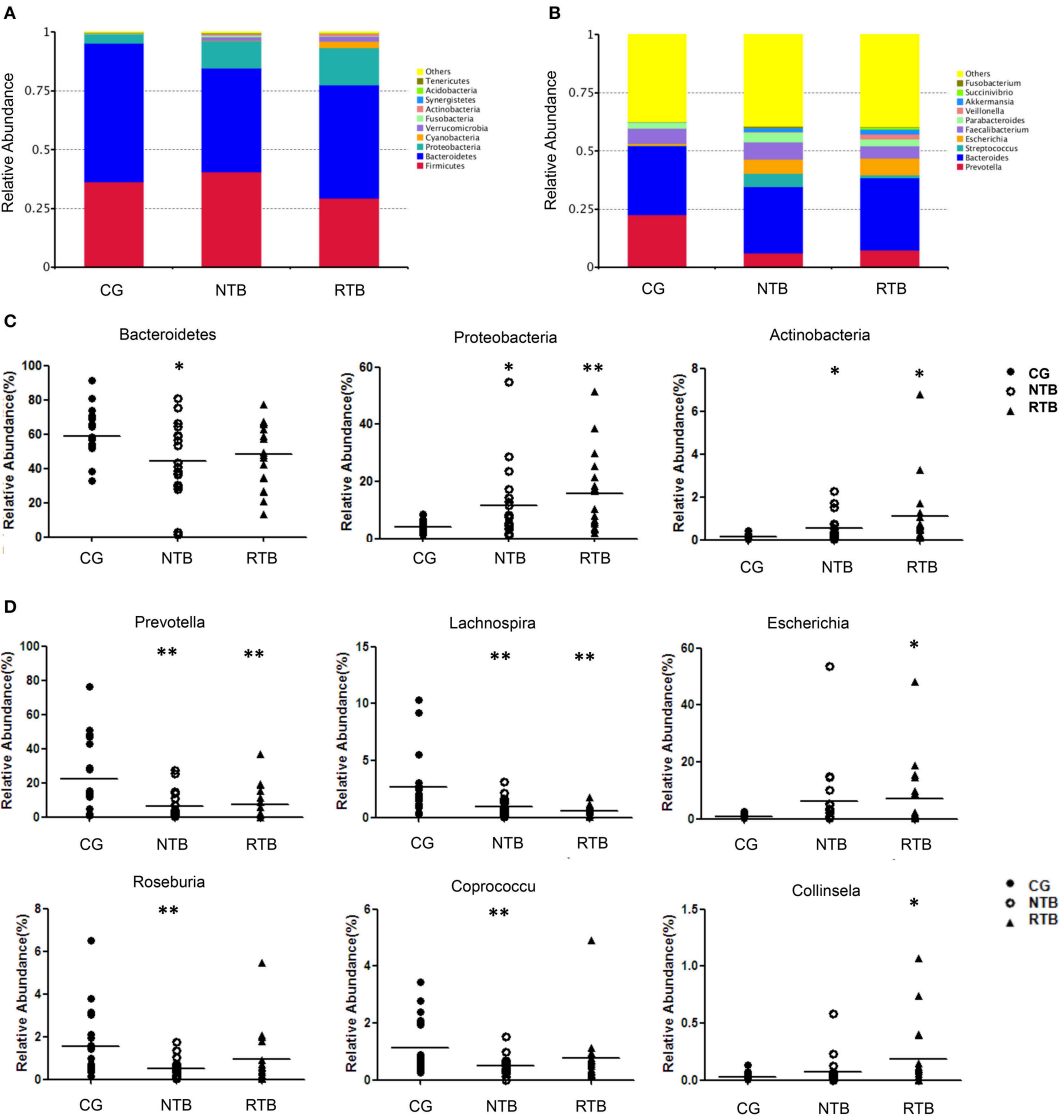
The major gut bacterial phyla and genus in new and recurrent tuberculosis patients as well as the healthy control. **(A)** Bacterial phylum levels of new and recurrent tuberculosis groups, NTB and RTB respectively. **(B)** Bacterial genus levels. **(C)** Comparison of gut microbiota of relative abundance at the bacterial phylum levels. **(D)** Comparison of gut microbiota of relative abundance at genus levels. The *p*-values were calculated using *T*-test, and significance was compared against the CG. ^*^P < 0.05. ^**^P < 0.01.

Among the 10 most abundant bacterial phyla, the relative abundance of the phyla Bacteroidetes, which is the largest component of gut microbe with more than 50% of the total abundance and contains many beneficial commensal organisms, was decreased in the two groups of tuberculosis in comparison with the healthy controls (Figure 3A). Firmicutes, the second largest phylum of gut microbe, was moderately decreased in the RTB, but was without significant change between the new group and the control group. Importantly, phylum Proteobacteria, which contain many Gram-negative bacteria and opportunistic pathogenic species, was significantly increased 2-fold in the new group, and 4-fold in the RTB in comparison with that in the control group (CG). At the bacterial genus level (Figure 3B), *Bacteroides* was without significant difference between the two tuberculosis groups (29.6% in CG, 28.4% in NTB, 31.0% in RTB). Genus *Prevotella* was significantly decreased in the two tuberculosis groups (22.5% in CG, 6.2% in NTB, 7.3% in RTB). Conversely, genera *Escherichia* was massively increased in both tuberculosis groups (0.8% in CG, 6.3% in NTB, 7.1% in RTB); and *Streptococcus* gave a similarly increased in the two tuberculosis groups (0.2% in CG, 5.6% in NTB, 1.4% in RTB).

To further understand the microbial communities among those groups, we compared the relative abundance at the bacterial phyla and genus levels among the three groups. At the bacterial phyla levels, the NTB (Figure 3C) demonstrated a marked decrease of *Bacteroidetes* in comparison with the control group (44.1 vs. 58.8%, *p* = 0.020). However, the phylum *Proteobacteria* was significantly enriched in the two tuberculosis groups: 11.7% of *Proteobacteria* for NTB vs. 4.0% for the CG, (*p* = 0.017), and 15.8% of *Proteobacteria* in the RTB vs. 4.0% in the CG, (*p* = 0.001). Likewise, a similar trend was observed for phylum *Actinobacteria* in the NTB (0.5% NTB vs. 0.2% CG, *p* = 0.015) and the RTB (4.0% vs. 1.1%, *p* = 0.021).

As shown in the Figure 3D, the bacterial communities were further compared at the genus level. Genus *Prevotella*, which is affiliated with the phylum Bacteroidetes, was significantly decreased in the NTB over the control (6.2% NTB vs. 22.5% CG, *p* = 0.005); and the patients with RTB (7.3% vs. 22.5%, *p* = 0.010). Genus *Lachnospira* within the phylum Firmicutes was markedly decreased in the NTB (0.9% NTB vs. 2.7%CG, *p* = 0.010) and in the recurrent group compared with healthy control group (0.6% RTB vs. 2.7% CG, *p* = 0.003). The relative abundance of *Roseburia* within the phylum Firmicutes, which is a group of major “short chain fatty acids” (SCFA) producing microbe, was significantly reduced in the new tuberculosis (0.5% NTB vs. 1.5% CG, *p* = 0.008). SCFA are well known for their functions in innate immunity and energy homeostasis (Schauber et al., 2003; Lu et al., 2016). Coprococcus, also within the phylum Firmicutes, was mostly depleted in the patients in the new group compared with the control group (0.5% NTB vs. 1.1% CG, *p* = 0.012). However, genus *Escherichia* within the phylum *Proteobacteria* (7.0% RTB vs. 0.1% CG, *p* = 0.041) and *Collinsella* (0.1% RTB vs. 0.03% CG, *p* = 0.042) within the phylum *Actinobacteria*, were enriched in the recurrent group. Nevertheless, bacteria groups in Figure 3D did not show any statistical difference between the two tuberculosis groups.

### Gut bacteria as biomarkers in the new and recurrent tuberculosis

**Figure 4:**
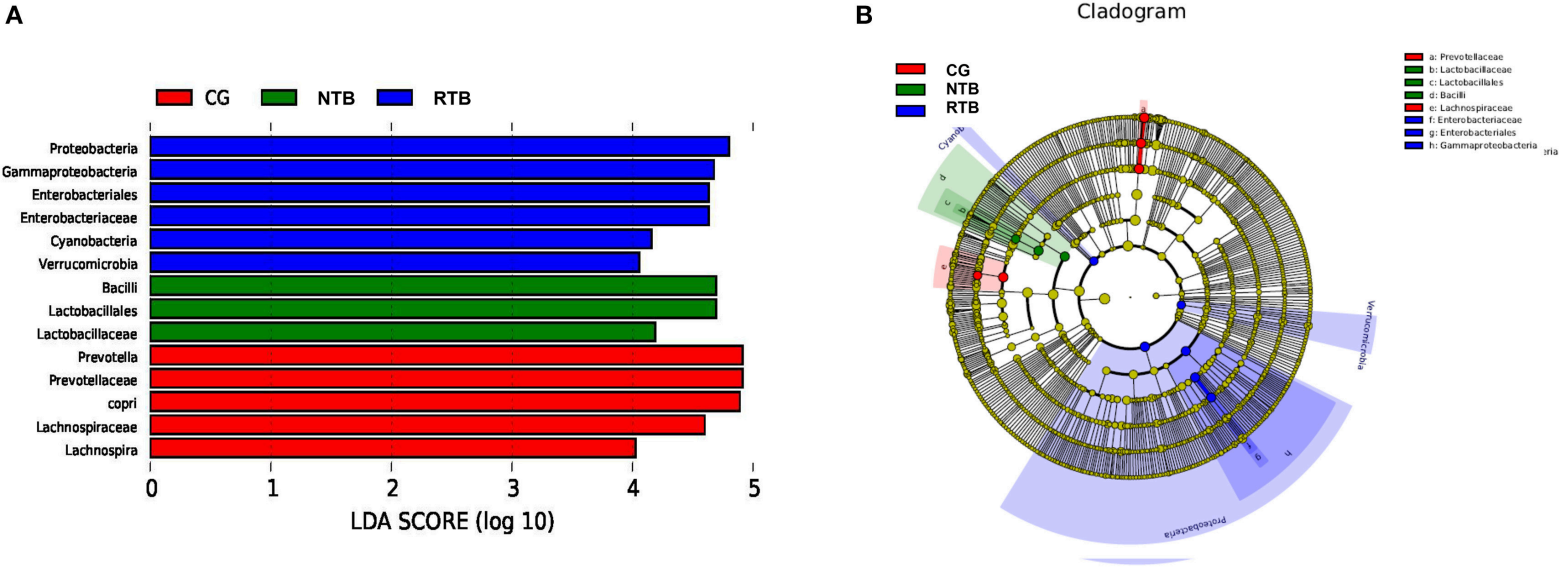
Different species as biomarkers in relative abundance identified by LEfSe analysis between the two groups of tuberculosis patients and the healthy control. **(A)** The length of the column represents the influence of significantly different species in relative abundance (LDA scores > 4). **(B)** The significantly different species are shown in the clado-gram. Each circle represents phylogenetic level from phylum to genus inside to outside. Each circle's diameter is proportional to the taxon's abundance and biomarker is consistent with the group marked with color. Red in CG, green in NTB, and blue in RTB.

To identify key biomarkers differentially displayed between the two tuberculosis groups, we performed a statistical analysis using LEfSe analysis with LDA to characterize different species between the groups. A total of 14 taxa were identified that were significantly different between the groups (Figure 4). The recurrent group (RTB) showed the most unique microbiota by a higher abundance of Proteobacteria (phylum), Gamma-Proteobacteria (class within Proteobacteria), Enterobacteriales (order within Proteobacteria), Enterobacteriaceae (order within Proteobacteria), Cyanobacteria (phylum) and Verrucomicrobia (phylum). We found that the abundance of family *Bacilli–Lactobacillales-Lactobacillaceae* was higher in the two tuberculosis groups.

### Relative abundance of genus *Prevotella* and *Lachnospira* was associated with patient's peripheral CD4^+^ T cell counts

**Figure 5:**
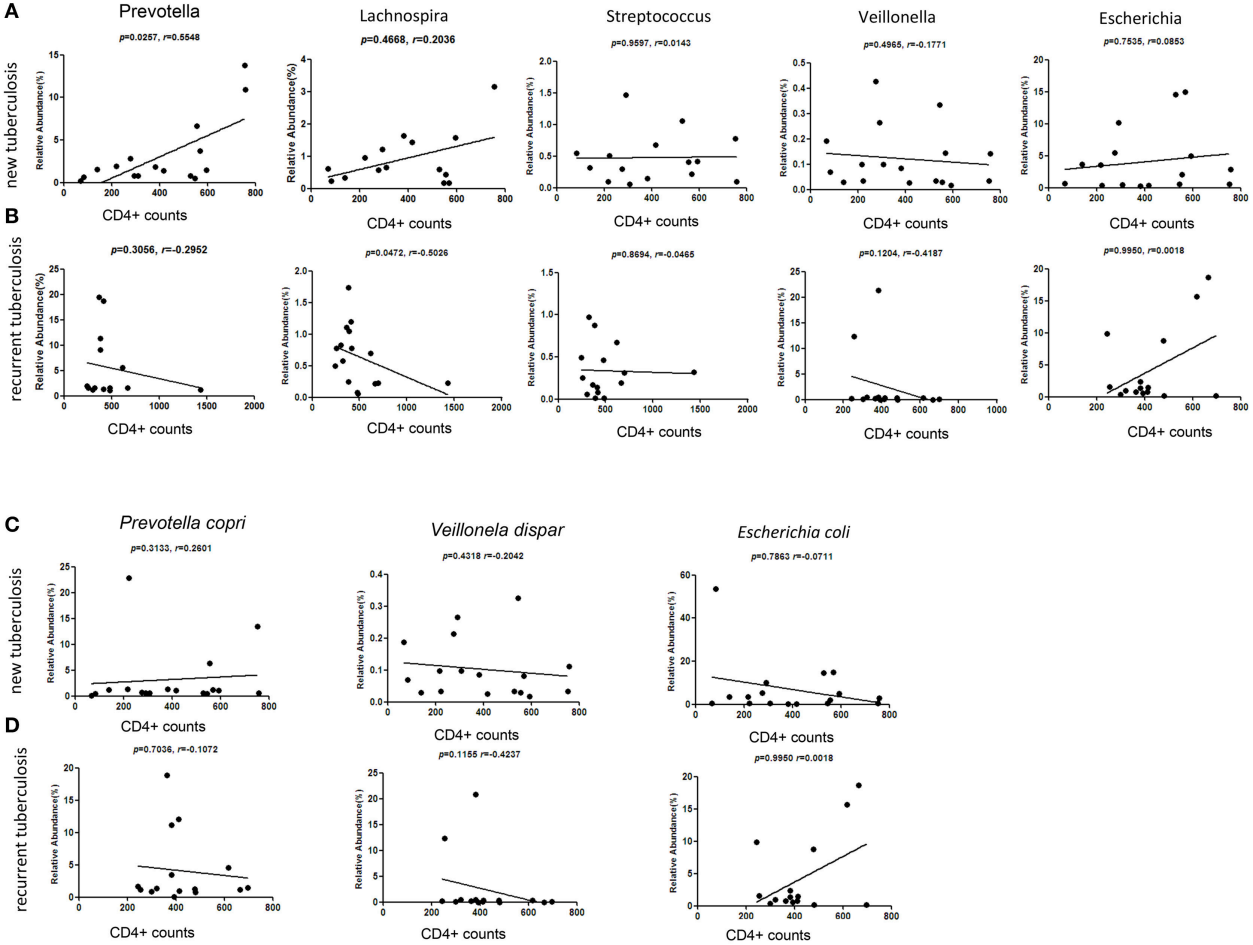
Correlation between the relative abundance of *Prevotella, Lachnospira, Streptococcus, Veillonella, Escherichia* with blood CD4^+^ T cell counts. Taxa of bacteria at the genus level were further analyzed at each of patient group. **(A,C)** The new tuberculosis group. **(B,D)** The recurrent tuberculosis group. X axis denotes the CD4^+^ T cell counts/μl. The assessment was done with Spearman's rank order correlation. Black dots indicate data.

CD4+ lymphocytes are the major adaptive immune cells for infective responsiveness. We explored the potential association between the fecal microbiota and CD4+ cells in the two types of tuberculosis. We applied nonparametric Spearman's rank correlation coefficients to measure the relative abundance of most enriched and least enriched bacterial taxa with the peripheral CD4^+^ cell counts in the two types of patients. Provotella, Gram-negative bacteria that are members of the oral and vaginal flora, are often recovered from anaerobic infections of the respiratory tract. We found that Prevotella at the genus level (*p* = 0.025) were strongly and positively correlated with CD4^+^ cell counts in patients with NTB (Figure 5A). Conversely, in the RTB, the gut Prevotella levels exhibited a negative association with CD4^+^ cell counts (Figure 5B).

Gut *Lachnospira* was positively related to CD4+ counts in the NTB, but was negatively related to CD4+ counts in the RTB (Figures 5A,B). Genus *Streptococcus, Veillonella* and *Escherichia* showed no clear correlation with the CD4^+^ cells in the tuberculosis patients. We also analyzed the three bacterial taxa, which are indicative of dysbiosis at the species levels, *Veillonella dispar, Prevolellacopri, Escherichia coli*, and none of them showed reliable correlation with CD4^+^ cell counts (Figures 5C,D).

## Discussion

The gut microbiota, through intimate interaction with host intestinal epithelial cells, co-evolves with the host. To some extent, the gut microbiota is regarded as an essential “organ” for the host. For example, gut microbes as an antigen source configure the early age development of the immune system for the host. The enterotype of gut microbiota is influenced by genetic and epigenetic factors from the host, the dietary and nutrition composition, and environmental factors. Gut microbes are known a major source of vitamin B series which serve as the co-factors in metabolic pathways. On the other hand, commensal microbiota, through production of antagonizing agents such as lantibiotics, can efficiently expel foreign invasion from the host. In return, the host provides protection for the beneficial symbiotic gut microbes. For instance, we recently demonstrated that vitamin D signaling through regulation of Paneth cell alpha-defensins promotes gut microbiota eubiosis, which consequently inhibits the high-fat-diet-induced metabolic disorders and fatty liver (Su et al., 2016).

*M. tuberculosis* has a great capacity to escape immune surveillance and can promote immune tolerant granulomata to protect the pathogens from immune clearance. The majority of *M. tuberculosis* exposed individuals remain asymptomatic but exhibit varying levels of immunity against *M. Tuberculosis* infection, indicating host factors determining the outcomes of the infection. In an animal model, one recent report showed changing gut microbiota during the *M. Tuberculosis* infection (Winglee et al., 2014). Also, in a murine infection model, another recent report showed that the oral antibiotic-mediated gut dysbiosis could promote *M. tuberculosis* infection and dissemination (Khan et al., 2016). Conversely, the report showed that fecal transplant of microbes from the control mice attenuated *M. tuberculosis* infection and improve prognosis. Short chain fatty acids (SCFAs), as a major group of metabolites from gut microbes, are essential for host immunity such as Treg induction and protection of gut epithelia, in addition to providing an energy source (Lin et al., 2015; Asarat et al., 2016; Correa-Oliveira et al., 2016). One report showed butyrate could antagonize the *M. tuberculosis*-induced pro-inflammatory response in macrophages and concomitantly promote IL-10 expression (Lachmandas et al., 2016). However, clinical evidence remains largely unknown for the association between gut microbiota and *M. tuberculosis* infection, prognosis, and recurrence. A recent report showed that colonization with Helicobacter is concomitant with modified gut microbiota and drastic failure of the immune control of *Mycobacterium tuberculosis* (Majlessi et al., 2017).

In the present study, through 16S rDNA gene sequencing analysis we initially measured gut bacterial communities in new and recurrence of tuberculosis infections; and associate gut microbiota with *M. tuberculosis* infection and recurrence. Our results showed that the gut microbiota are significantly different in the two types of tuberculosis and the healthy control groups. We further found that specific changes in the gut microbiota were associated with patient's peripheral CD4^+^ T cell counts. To our knowledge, this is the first documentation of changing gut microbiota with the *M. tuberculosis* infection and recurrence.

Specifically, we found the ACE-diversity index was increased significantly in the recurrent tuberculosis; and the un-weighted unifracPCoA in that group was notably different from the control group. In agreement with our finding, one report showed that the microbiota in the sputum of pulmonary tuberculosis patients were more diverse than those of healthy participants (Cui et al., 2012). Furthermore, in the sputum, many foreign bacteria were unique to pulmonary tuberculosis patients. Another study found that Proteobacteria and Bacteroidetes were more represented in the sputum of tuberculosis samples, while Firmicutes was more predominant in the controls (Cheung et al., 2013).

We also investigated the gut microbial composition at different phylogenic levels for their relationship with the tuberculosis groups. We found that Proteobacteria and Actinobacteria were enriched, while Bacteroidetes was mostly depleted at the phyla levels in the patients with tuberculosis recurrence. This change is consistent with previous studies conducted in sputum (Wu et al., 2013). Genus Prevotella, which is affiliated with the phylum *Bacteroidetes* and genus *Lachnospira* within the phylum *Firmicutes* were significantly decreased in both tuberculosis patient groups. The genus Lachnospiras and Roseburia, both within the phylum Firmicutes, have been known for their production of short-chain fatty acids (SCFAs) (Duncan et al., 2007; Berni Canani et al., 2012). Here, we noticed the reduction of genus Lachnospiras and Roseburiain in the two groups of tuberculosis patients, indicating potential impairment of SCFA production and the consequent metabolic disorders, which may further impact on the tuberculosis infection and prognosis, a topic under our investigation (Supplementary Figure S1). The functions of butyrate are known for promoting intestinal health in part through maintaining the integrity of the intestinal mucosa (Roy et al., 2006) and regulation of host immune response (Maslowski et al., 2009). We also found that *Escherichia coli*, which is affiliated with the Proteobacteria was enriched in patients with RTB. Interestingly, the genus *Prevotella* was decreased in both tuberculosis groups.

Another interesting finding was that Prevotella positively correlated with the peripheral CD4^+^ cells in new tuberculosis cases, and inversely correlated with recurrent tuberculosis cases. A similar trend was found for Lachnospira. These data suggest a new hypothesis that specific gut microbes may associate with host's immune status and related to prognosis and outcome of patients. The specific gut microbes in the tuberculosis patients need to be further identified and characterized as potential biomarkers for the tuberculosis prognosis.

Type-2 diabetes mellitus confers a 3-fold of increased risk for tuberculosis. How diabetes promotes *M. tuberculosis* infection and dissemination is a big clinical challenge, and gut microbes could be the key mediators between diabetes and tuberculosis. Increased phylum of Proteobacteria and decreased phylum of Bacteroidetes had been well documented in diabetes and obesity (Ley et al., 2005; Turnbaugh et al., 2009). One recent study showed the influence of physiological concentrations of butyrate on cytokine responses to *M. tuberculosis* in human peripheral blood mononuclear cells (PBMCs) (Lachmandas et al., 2016). The study found that butyrate suppressed the tuberculosis-induced proinflammatory cytokine responses, while it increased production of IL-10, an immune regulatory cytokine. Altered gut microbiota may also contribute to the recurrence of tuberculosis. Recurrent tuberculosis creates substantial burden for tuberculosis, and it may arise through relapse of the original infection or through reinfection with a new strain. High rate of relapse is often associated with unsuccessful treatment and drug resistance. A high rate of recurrence due to reinfection implies a failure to develop protective immunity after the first episode and is often related to human immunodeficiency virus infection, while other factors may contribute as well. Whether and how the altered gut microbiota contributes to recurrence is an urgent problem that requires attention.

This initial clinical study should be extended and refined. First, a major and common challenge for a clinical study shared by the current one is to recruit the participants with similar backgrounds and characteristics. Another common limitation shared by our study is the small number of human subjects. Our results will require validation in larger cohort studies.

## Author contributions

ML and PW conducted the experimental work. YL, D-XL, QS, HZ, Q-FL, and RH participated in discussion and data analysis. SP, Y-PH, and YZ conceived the project and wrote the paper.

### Conflict of interest statement

The authors declare that the research was conducted in the absence of any commercial or financial relationships that could be construed as a potential conflict of interest.

## Abbreviations

MTB: *Mycobacterium tuberculosis*
NTB: new tuberculosis patients
RTB: recurrent tuberculosis patients
OTU: operational taxonomic unit
PCA: principal component analysis
PCR: polymerase chain reaction.
