# Supplementary material for: Alternation of Gut Microbiota in Patients with Pulmonary Tuberculosis
Source: Front Physiol. 2017 Nov 17;8:822. doi: 10.3389/fphys.2017.00822 (PMC5698276; doi:10.3389/fphys.2017.00822)

Supplementary

Changing Gut Microbiota in Patients with Pulmonary Tuberculosis

Mei Luo[1,2], Yong Liu[2], Pengfei Wu[1], Dong-Xia Luo[2], Qun Sun[1], Han Zheng[1], Richard Hu[3], Stephen J. Pandol[4] and Yuan-Ping Han[1,*] and Yilan Zeng[2,*]

Figure Major butyrate producing bacteria

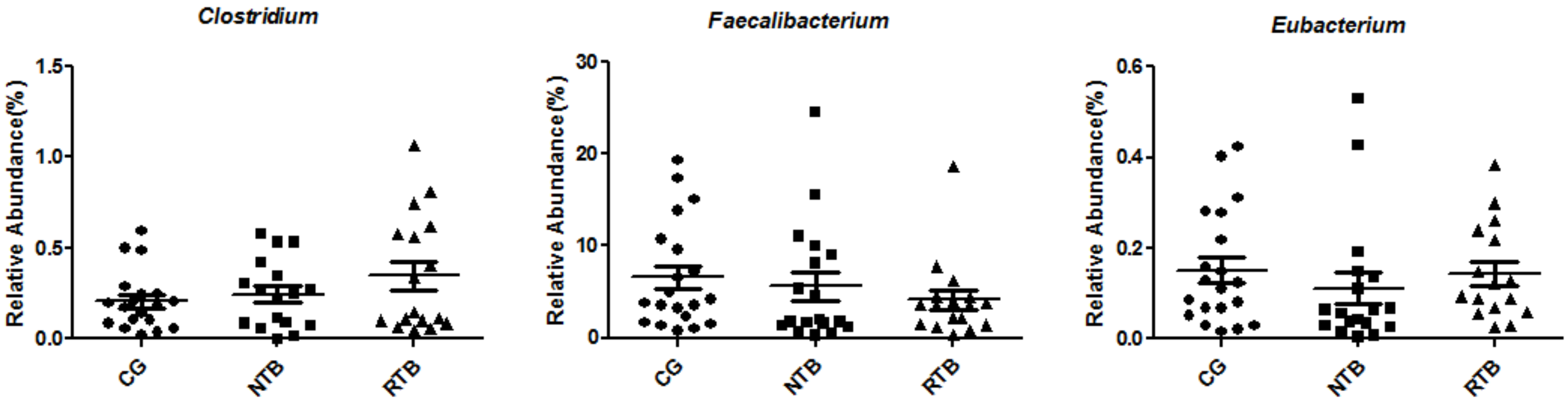

Supplementary Species-t test

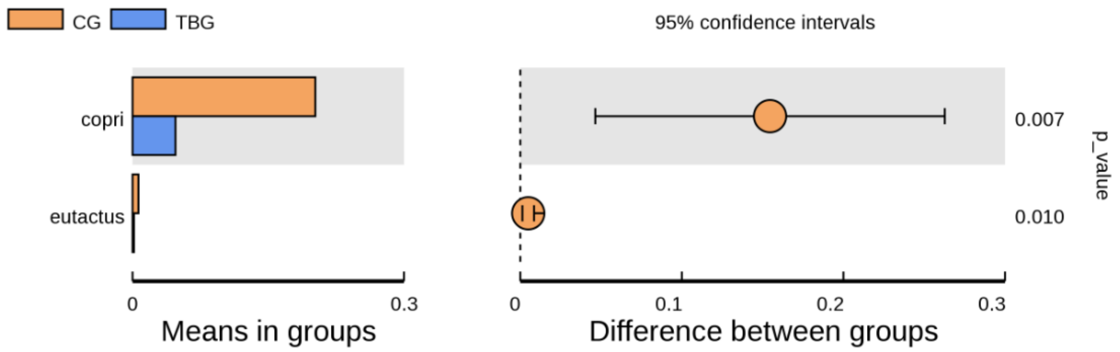

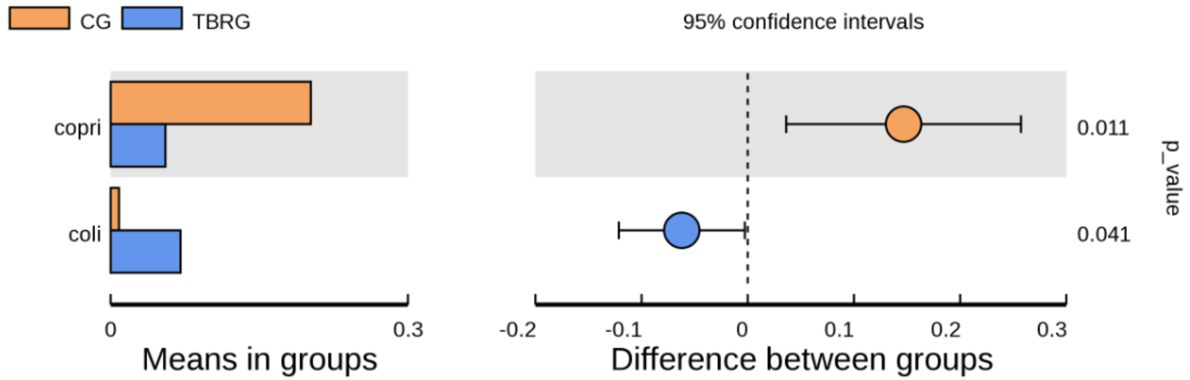

Supplement: Supplementary file 1 [file Presentation1.PDF]
